# Supplementary material for: Molecular and Morphological Assessment of Septoria Species Associated with Ornamental Plants in Yunnan Province, China
Source: J Fungi (Basel). 2021 Jun 16;7(6):483. doi: 10.3390/jof7060483 (PMC8234678; doi:10.3390/jof7060483)
Supplement: Supplementary file 1 [file jof-07-00483-s001.zip › Supl Table S1.pdf]

Supl. Table S1. DNA base difference between our *Septoria* strains and related species.

Species	Strain number	TEF (1-393bp)	RPB2 (394-736bp)	TUB (737-1049bp)	ITS (1050-1530bp)	LSU (1531-2329bp)
S. sanguisorbigena	GUCC 2131.2	0	0	0	2	0
S. sanguisorbigena	GUCC 2164.1	0	0	0	2	0
S. sanguisorbigena	GUCC 2164.2 T	0	0	0	0	0
S. posoniensis	CBS 128645	4	7	7	0	0
S. exotica	CBS 163.78	6	2	6	2	0
S. chrysanthemella	CBS 128716	21	13	13	2	3
Species	Strain number	TEF (1-393bp)	RPB2 (394-736bp)	TUB (737-1049bp)	ITS (1050-1533bp)	LSU (1534-2332bp)
S. pileicola	GUCC 2131.3	0	0	0	1	0
S. pileicola	GUCC 2131.4 T	0	0	0	0	0
S. posoniensis	CBS 128645	4	8	0	3	0
S. exotica	CBS 163.78	6	3	9	3	0
S. chrysanthemella	CBS 128716	20	14	16	3	3
S. sanguisorbigena	GUCC 2164.2 T	0	1	7	1	0
Species	Strain number	TEF (1-393bp)	RPB2 (394-736bp)	TUB (737-1049bp)	ITS (1050-1530bp)	LSU (1531-2329bp)
S. longipes	GUCC 2131.1 T	/	0	0	0	0
S. posoniensis	CBS 128645	/	5	8	2	0
S. exotica	CBS 163.78	/	5	7	2	0
S. chrysanthemella	CBS 128716	/	14	14	2	3

Species	Strain number	TEF (1-387bp)	RPB2 (388-727bp)	TUB (728-1038bp)	ITS (1039-1493bp)	LSU (1494-2292bp)
S. protearum	GUCC 2127.3	0	0	0	0	0
S. protearum	CBS 778.97 T	0	0	0	0	0
Species	Strain number	TEF (1-393bp)	RPB2 (394-736bp)	TUB (737-1046bp)	ITS (1047-1540bp)	LSU (1541-2339bp)
S. dispori	GUCC 2164.3	0	0	0	0	0
S. dispori	GUCC 2164.4	0	0	0	0	0
S. dispori	GUCC 2127.1 T	0	3	0	4	0
S. verbenae	CBS 113438	54	15	23	0	6
S. coprosmae	CBS 113391	43	43	23	1	0
Species	Strain number	TEF (1-393bp)	RPB2 (394-736bp)	TUB (737-1046bp)	ITS (1047-1501bp)	LSU (15015-2300bp)
S. dispori	GUCC 2127.4	0	0	0	0	0
S. verbenae	CBS 113438	54	16	25	0	6
S. coprosmae	CBS 113391	43	43	16	0	0
S. dispori	GUCC 2164.3 T	0	1	11	0	0
Species	Strain number	TEF (1-388bp)	RPB2 (389-732bp)	TUB (733-1041bp)	ITS (1042-1526bp)	LSU (1527-2325bp)
S. protearum	CBS 778.97 T	0	0	0	0	0
S. critri	CBS 315.37	1	0	/	0	0
S. criticola	CBS 356.36 T	1	1	0	0	0
S. limonum	CBS 419.51	1	0	0	0	0
S. chamaecisti	CBS 350.58	0	0	1	0	0

S. gerberae	CBS 410.61	0	11	0	1	0
S. hederae	CBS 566.88	0	10	0	0	0
S. lobeliae	CBS 113392	0	9	0	1	0
