# Supplementary figures and images for: Molecular and Morphological Assessment of Septoria Species Associated with Ornamental Plants in Yunnan Province, China
Source: J Fungi (Basel). 2021 Jun 16;7(6):483. doi: 10.3390/jof7060483 (PMC8234678; doi:10.3390/jof7060483)

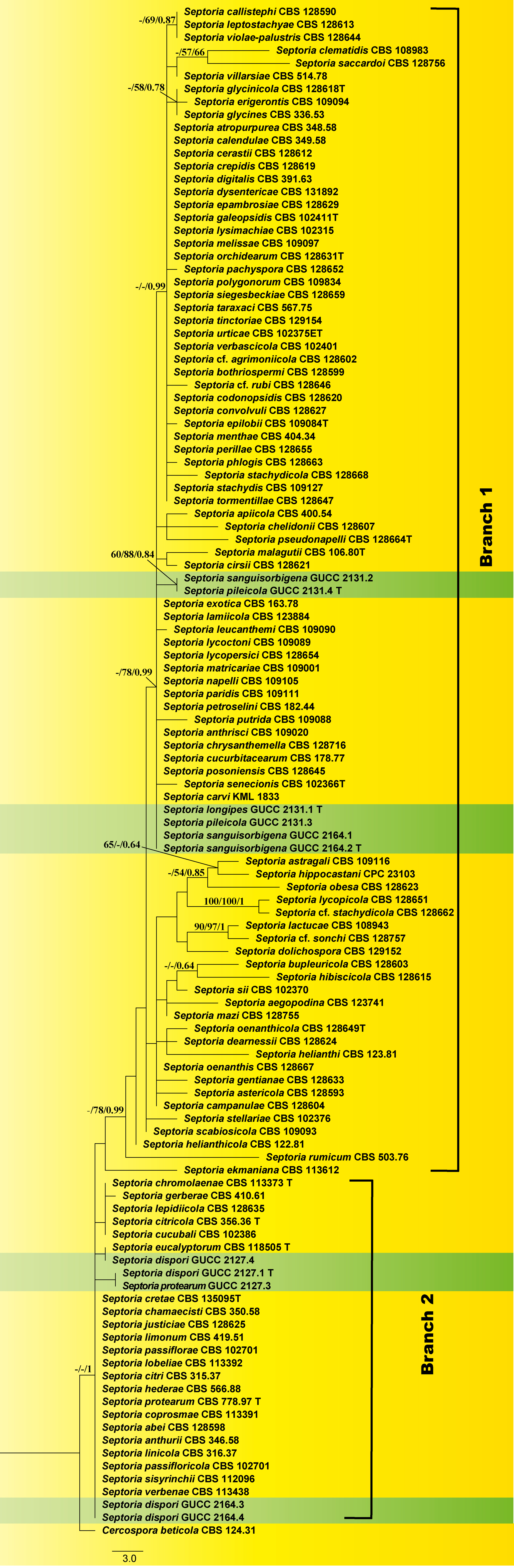

Supplement: Supplementary file 1 [file jof-07-00483-s001.zip › Sup. Figure S1.jpg]

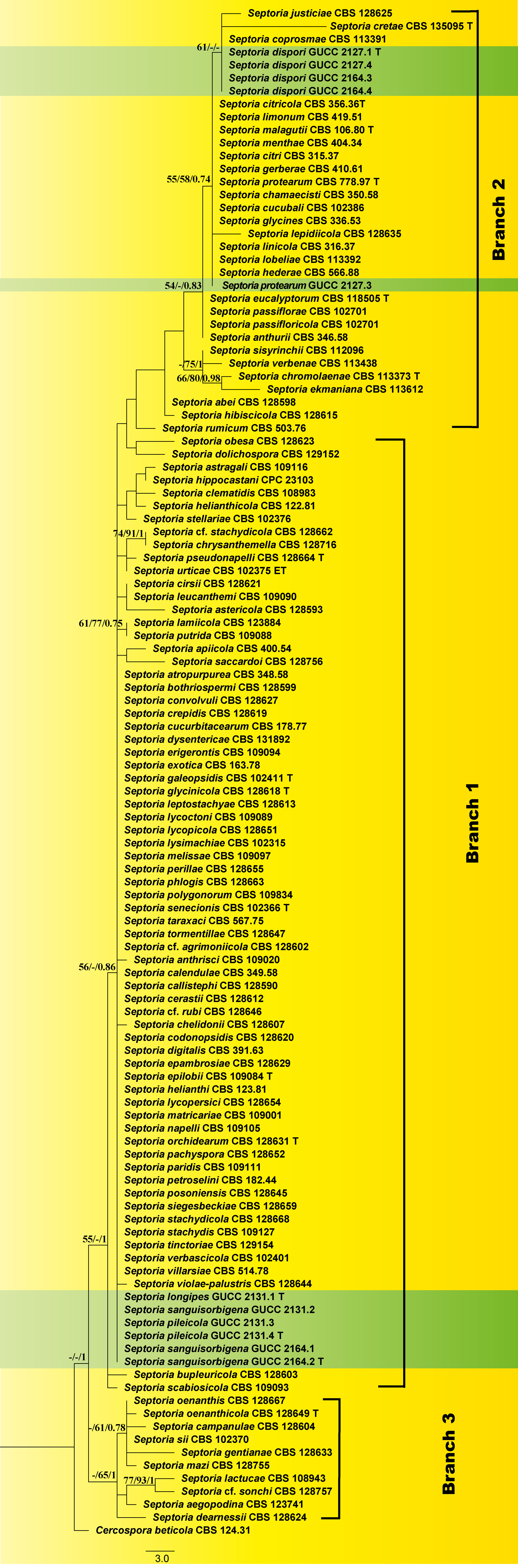

Supplement: Supplementary file 1 [file jof-07-00483-s001.zip › Sup. Figure S2.jpg]

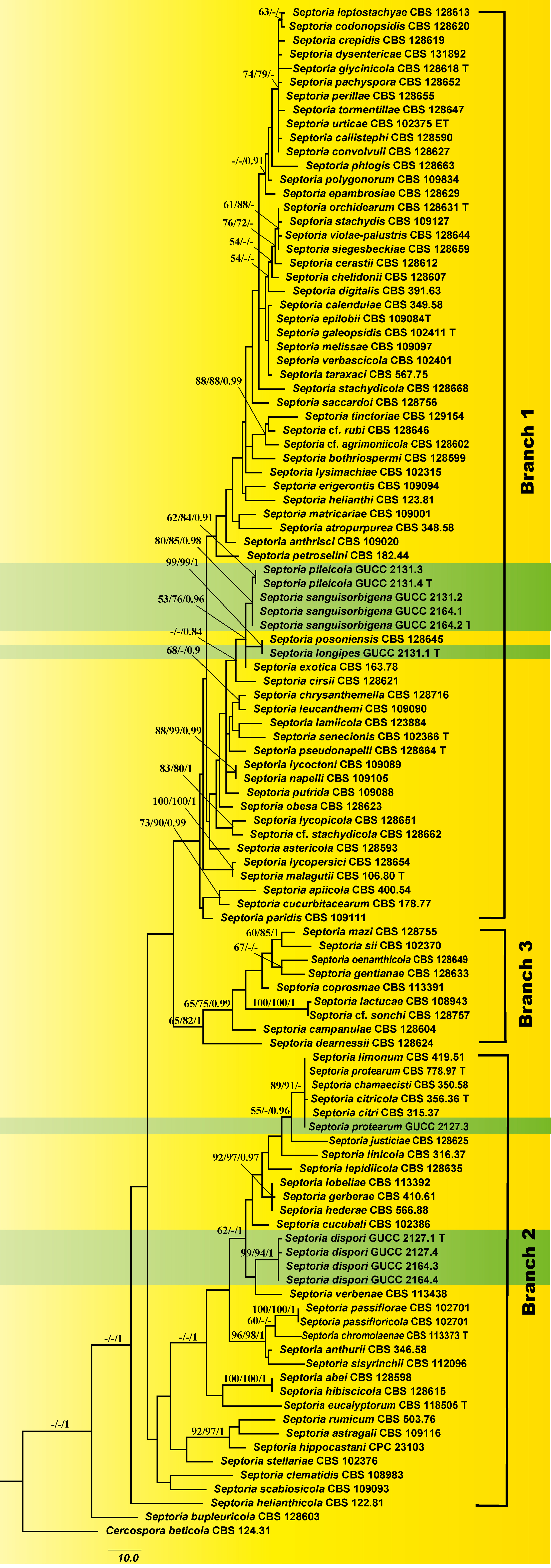

Supplement: Supplementary file 1 [file jof-07-00483-s001.zip › Sup. Figure S3.jpg]

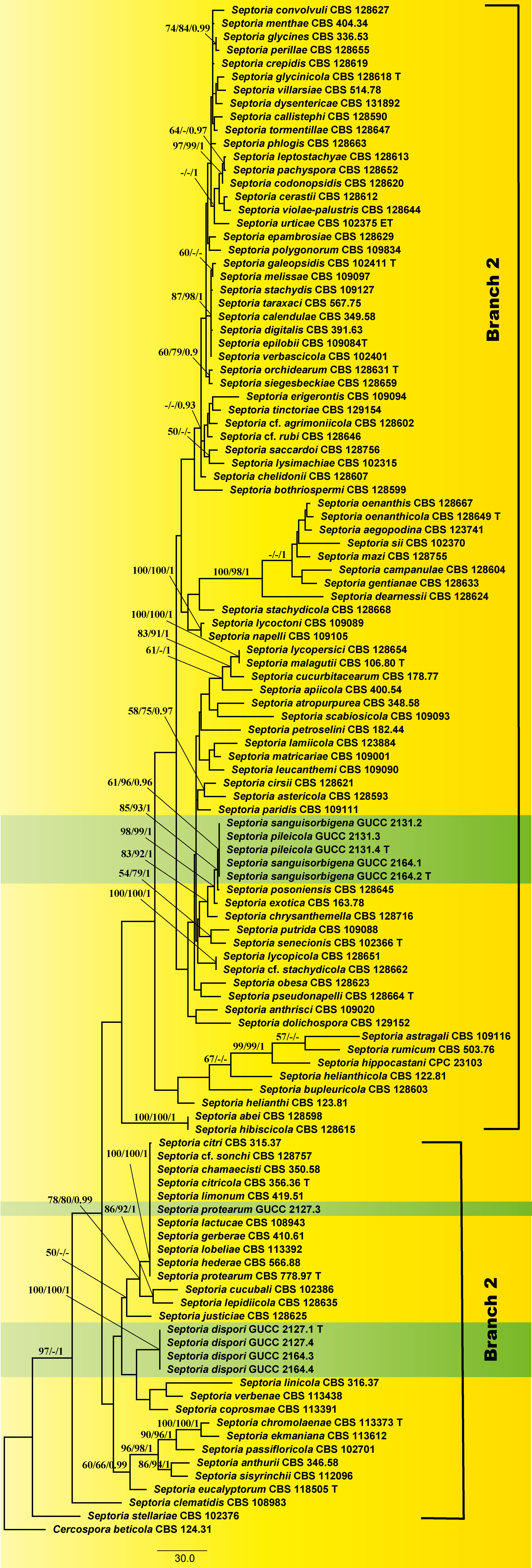

Supplement: Supplementary file 1 [file jof-07-00483-s001.zip › Sup. Figure S4.jpg]

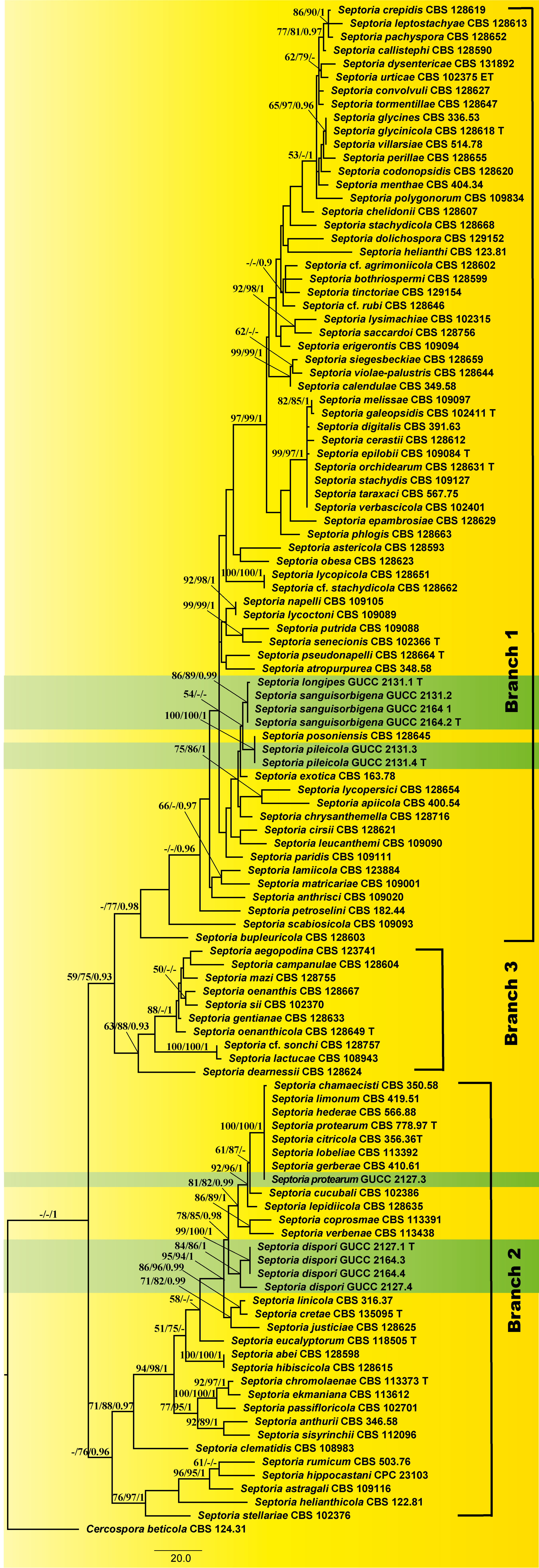

Supplement: Supplementary file 1 [file jof-07-00483-s001.zip › Sup. Figure S5.jpg]
